# Finite element models of intervertebral disc: recent advances and prospects

**DOI:** 10.1080/07853890.2025.2453089

**Published:** 2025-01-22

**Authors:** Tianze Sun, Junlin Wang, Xin Liu, Huagui Huang, Jinzuo Wang, Moran Suo, Jing Zhang, Zhonghai Li

**Affiliations:** a Department of Orthopedics, First Affiliated Hospital of Dalian Medical University, Dalian, The People’s Republic of China; b Key Laboratory of Molecular Mechanism for Repair and Remodeling of Orthopedic Diseases, Liaoning Province, Dalian, The People’s Republic of China

**Keywords:** Intervertebral disc, finite element, modeling, application, biomechanics analysis

## Abstract

**Objectives:**

The incidence rate of intervertebral disc degeneration (IVDD) is increasing year by year, which brings great harm to our health. The change of biomechanical factors is an important reason for IVDD. Therefore, more and more studies use finite element (FE) models to analyze the biomechanics of spine.

**Methods:**

In this review, literatures which reported the FE model of intervertebral disc (IVD) were reviewed. We summarized the types and constructional methods of the FE models and analyzed the applications of some representative FE models.

**Results:**

The most widely used model was the nonlinear model which considers the behavior of porous elastic materials. As more advanced methods, More and more models which involve penetration parameters were used to simulate the biological behavior and biomechanical properties of IVD.

**Conclusions:**

Personalized modeling should be carried out in order to better provide accurate basis for the diagnosis and treatment of the disease. In addition, microstructure, cell behavior and complex load should be considered in the process of model construction to build a more realistic model.

## KEY MESSAGES

The nonlinear models in the primary stage considered the behavior of porous elastic materials, but rarely consider the effect of osmotic pressure on liquidity.

The advanced multiphase models considering permeability parameters have been applied widely during the construction of FE models of IVDs.

In the future, realistic models should make reliable connections between the multi-scale models and microstructures under more complex mechanical loading conditions.

## Introduction

With the aging population and increased attention towards non-communicable diseases, the global incidence of low back pain has been increasing annually, as indicated by the number of medical treatments, and it is the main cause of lost work. Low back pain is a complex symptom of disability and pain that is caused by many factors including psychological factors, biomechanical factors, and pain-processing mechanisms [1,2]. Back pain has become a globally common symptom that occurs in all age groups, from children to the elderly, especially in low income and middle-income populations. Nearly 50% of people 70 years old are affected by low back pain, and disability-adjusted life-years characteristic changed by 46.9% from 1990 to 2019 [3–5]. Intervertebral disc degeneration (IVDD) is one of the different types of the pain and the integrity of intervertebral disc (IVD) is affected by many factors, such as genetic, mechanical traumatic factors, and hormones [6,7]. As the height of the IVD decreases, there are changes in the dynamics of the associated segment of the spine, which narrows the spinal canal and compresses the nerve, resulting in low back pain [8].

Because of ethical limitations using human samples and the technical complexities of body measurements, the finite element method (FEM) has been used as a cost-effective tool to study IVD. In 1972, FEM was first used in the analysis of biomechanics to evaluate the external and internal response of experimental tissues [9]. FEM was quickly then applied to investigate the spine to quantitatively and qualitatively study IVDD. FE analysis evaluates the complex biological, mechanical information of discs under all conditions, including physiological and pathological that are quite difficult for traditional methods. However, measurements only cannot clarify the correlation between IVDD and mechanical loads. Accordingly, a finite element (FE) model was developed to supplement studies of IVDD, especially to understand the relationships between the biomechanical performance and degeneration of IVD. An advantage of the FE model is the ability to evaluate the effect on the final results by altering any input factor. It is also able to predict the initiation and progression of failure because of cyclic loading in lumbar segments [10]. Some early models only use the porous elastic properties of the segment to describe the deformation and flow of the fluid in IVD. More porous elastic FE models of disc tissues include strain-free swelling pressure and permeability [11]. These improvements help us understand the biomechanical changes of the spine during cyclic loading and the combined effect of moment loadings.

The purpose of this narrative review is to summarize the types and applications of FE models of the IVD and the advantages and disadvantages of various models. We aimed to provide a new writing idea for similar articles and research. We also hope to provide a theoretical guidance for etiological analysis, selection of treatment methods, and predicting the prognosis of IVDD.

## Method

### Study design

The aim of this study was to review intervertebral disc finite element modelling and to discuss recent advances, prospects and limitations of intervertebral disc finite element modelling, as well as the strengths and weaknesses of each model. The study was conducted through academic databases PubMed, Web of Science, Google Scholar, Scopus, and related literature resources, and the following terms and their derivatives were considered during the search: finite element modelling (FEM), intervertebral disc, biomechanics, and applications. The aim was to summarise the differences and characteristics of each modelling approach, as well as the microstructures, cellular behaviours and complex loads that are considered during the modelling process, in order to build more realistic models. The aim is to summarise the differences and characteristics of each modelling approach, as well as the microstructures, cellular behaviours and complex loads considered during the construction process, so as to build more realistic models. It facilitates the selection of appropriate modelling methods and provides an accurate basis for the diagnosis and treatment of diseases.

### Eligibility criteria

Studies included in the review met the following criteria: (1) English-language articles; (2) finite element modelling of the intervertebral disc; and (3) adjunctive diagnosis or personalised treatment through intervertebral disc finite element modelling. However, studies that met any of the following criteria were excluded: (1) duplicate articles; (2) unrepresentative research; (3) articles presenting ideas or frameworks that have not yet been applied; (4) articles not based on finite element modelling of the intervertebral disc in medicine; (5) lack of finite element modelling techniques in building the intervertebral disc model; (6) articles published only in the form of conference abstracts; (7) proposals, protocols, letter, conference, or opinion type articles; (8) articles that apply some independent means of building disc models but do not integrate finite element techniques.

### Search results

**Figure 1.**
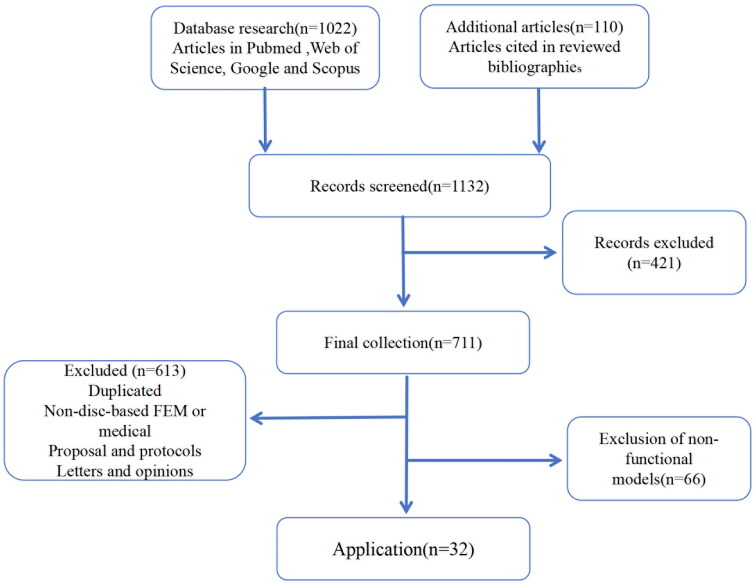
Flow diagram illustrating the screening process for papers in this study.

**Figure 2.**
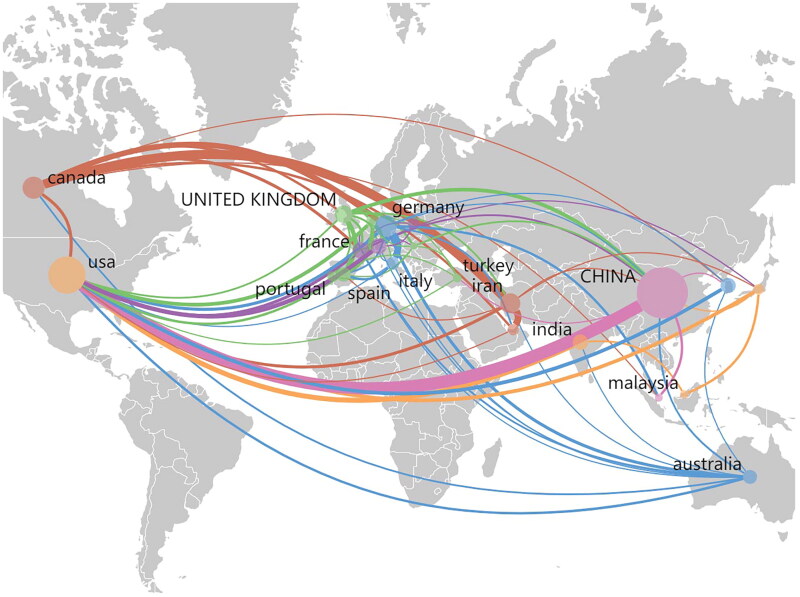
The situation of related articles published by various countries in the past twenty years.

**Figure 3.**
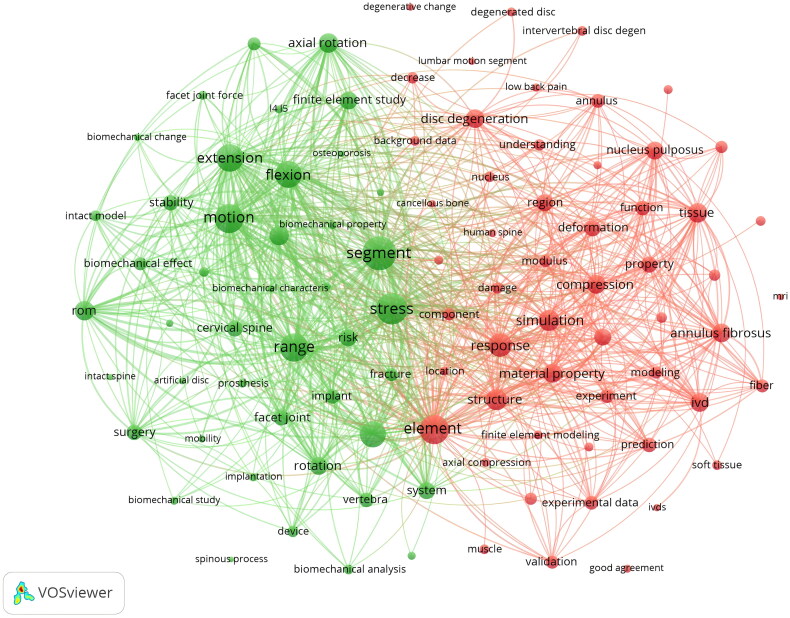
Keywords related to research hot spots in this field.

We retrieved a total of 1102 papers, of which 32 met the inclusion criteria and were therefore included. Met the inclusion criteria and were therefore included in further analysis. Screening procedure flow chart (Figure 1). Overall, the popularity of intervertebral disc finite element model research and application in the medical field is accelerating worldwide. We searched the publication of relevant articles in various countries over the past 20 years, with China and the United States having the most. Other countries such as Australia, Japan, Canada, Italy, and South Korea have also contributed to research in this field (Figure 2). And research hotspots in this field (Figure 3).

### Anatomy of IVD

The IVD comprises three main components: nucleus pulposus (NP), annulus fibrosus (AF), and cartilaginous endplates (CEP). The NP is mainly gelatinous proteoglycans that resist compression by maintaining spinal separation and the hydrostatic pressure. The AF is a tissue that surrounds the NP, which consists of oriented collagen fibers. The CEP is attached to the vertebrae and contains NP and AF inferiorly and superiorly, respectively. These components ensure the biomechanical stability of the spine. However, degeneration and injury of the disc lead to changes in the biomechanical properties, which in turn accelerate changes in the cell metabolism, nutrient supply, matrix metalloproteinase activity, and a variety of cytokines of IVD [12].

#### Nucleus pulposus

The NP is a gelatinous part of the IVD, consisting mainly of water (66–86%) and type II collagen. NP is essential for the mechanical flexibility of the spine [12]. The transport of metabolites and nutrients, such as lactic acid, glucose, and proteoglycan, plays an important role in the osmotic pressure of the IVD, and affects the biomechanical properties of the IVD [13–15]. The mechanical properties of the NP are fluid under low load rate compression and solid under a high load rate compression, and the mechanical properties of degenerative disc NP are also incline towards solid [16].

#### Annulus fibrous

The fiber ring comprises 15–25 concentric circular layers, each with a thickness less than 1 mm, surrounded by tough fiber tissue. The outer layer is mainly closely arranged type I collagen fibers that ensure smooth support of the tension and circumferential and axial pressure generated by internal nuclear pressure. The inner layer comprises type II collagen fibers that are combined with proteoglycan and water, allowing the tissue to withstand greater pressure [17]. The complete fiber ring structure helps to limit the NP and maintain the internal pressure of the IVD under load [18,19].

#### Cartilaginous endplates

The CEP is a layer of hyaline cartilage comprising water, type II collagen fibers, and proteoglycan, forming a three-dimensional network structure that helps to ensure material exchange, complete the pressure transfer between the NP and fibrous ring, and then transfer the pressure from the IVD to the vertebral body [20]. With age, calcification and pore blockage of the CEP will reduce its permeability and hinder the reabsorption of water and height recovery of the NP to change the biomechanics of IVD and reduce its load-bearing capacity [21,22].

### Biomechanics of IVD

#### Biomechanics of a normal IVD

The IVD has complex mechanics because of its inherent viscoelasticity, heterogeneous structure, and interstitial fluid–matrix interactions. In a healthy disc, the NP applies hydrostatic pressure to the AF fibers [23]. The stress and strain distribution of the lumbar is mainly concentrated on the posterior and bilateral posterior of the AF in a normal disc. For a normal disc under axial pressure, the range of the stress on the AF changes, and the intensity of the stress grows weaker from the AF to the NP [24]. The extrusive displacement of the posterior disc is the most obvious, which is consistent with clinical observations. Another experiment showed that the internal failure mechanism of the IVD varies with an increase in loading. In this experiment, the high-rate pulse loading caused the internal morphology of the IVD disruptions, which is significantly different from that caused by gradual low-rate loading [25]. Hydration and temperature both impact the biomechanical properties of the IVD [26].

During creep, the recovery deformations increase nonlinearly because of the substantial contribution of intrinsic viscoelasticity, porous elasticity, and elastic deformation during disc recovery [27].

#### Biomechanics of degenerated/injured IVD

The biomechanical properties of IVD are closely related to aging and degeneration, which give rise to systematic changes in intra disc stresses. A 3D volume reconstruction showed that a 26.6% decrease in disc height and a 20.6% increase in maximum transverse cross-section area leads to an 11.2% decrease in disc volume, which results in inhomogeneous stress distribution on the AF and lumbar instability [24,28]. With the aging and degeneration of the IVDs, the proteoglycan and water content in the NP decreases, becoming more solid like than liquid. The degenerated AF and CEP become calcified, which reduces the permeability of the IVD and transports metabolites [17].

IVDs normally carry part of the compressive load; when the spine is extended, the load the neural arch tissue bears is greater than when it is flexed, and the load bearing increases when the IVDs are narrowed because of sustained loading or degenerative changes [29]. In a complex posture, loading failure is determined by the loading rate, and the strain rate affects certain elements of IVDD [30]. The anterior rim lesion reduced the peak resistive moment, which was produced by the disc in extension, lateral bending, and axial rotation, and it also reduced the ability of the disc to resist motion [31].

### Application of FEM for IVD

The *in vitro* specimen testing method for biomechanical research has problems including cost, difficult technical realization, and limited research results [12]. FEM can solve these problems, and help researchers analyze and calculate the IVD internal stress changes. In the study of IVDD, FEM is a cost-effective alternative tool because it has the advantages of allowing repeated experiments, parameter variability, analyzing stress, and surgical design. With the improvements to computing power, FEM has been further applied to the study of the spine and IVDD, and has been developed from linear analysis to nonlinear model analysis, becoming a reliable research method. The validation of FE models is to demonstrate their relative bio-fidelity under similar conditions by comparison of its predictions with available measurements or clinical outcomes recorded in similar environments [32].

FEM can provide complex biological, chemical, mechanical, electrical information that is difficult to directly measure under the physiological and pathological conditions of the IVD. Therefore, FEM can be used to explore the etiology of the IVD, design and evaluate surgical instruments, and improve operation planning. FEM can help us fully understand the internal environment of the IVD and reveal the impact of compression on the nutrient distribution of the disc [33]. Dynamic compression can increase the oxygen concentration and reduce the accumulation of lactic acid, while static compression can increase the accumulation of lactic acid and reduce the oxygen concentration [34,35]. Another study showed that dynamic loading can significantly increase the solute transport through CEP compared with static loading, where the CEP with low porosity is more affected by dynamic loading [36]. FEM can also be used to clarify the impact of nutritional imbalance on the pathogenesis of IVDD. Studies have shown that damage to the CEP-NP pathway mainly causes degeneration of NP, damage to the AF pathway mainly contributes to the degeneration of the properties of outer AF, and simultaneous damage to the CEP-NP and AF pathways cause degeneration of NP and AF [37]. Cai et al. [38] established a spine model from CT scans of healthy men, and analyzed the biomechanical properties of the degenerated segment’s and adjacent segment’s discs by FEM. The study found that the degenerated IVD cause the movement and load of the adjacent normal segment, which may accelerate the degeneration of the IVD in the adjacent segment.

### Construction methods and tools for FE models

FEM is a powerful tool to study complex biomechanical characteristics of IVD at microscopic and macroscopic levels. Accordingly, FE models create a certain foundation for FEM and are reliable. Early models mimic the biomechanical properties of the IVD through simple geometry and mechanical behavior [39]. The most common model is the three-dimensional, porous–elastic nonlinear FE model, which couple biomechanical properties and biological properties using cell-activity coupled mechanical electrochemical theory and considers porosity, permeability, charge density, and solute diffusivity [37,40]. The anatomical properties of the IVD are obtained by computed tomography (CT) or magnetic resonance imaging (MRI) where the area is identified by landmarks and the FE mesh is morphed into a geometry [41]. Alternatively, FE parameters can be defined for modeling based on sample-averaged literature data. The degenerated IVD is simulated by reverse engineering of changing parameters on the basis of a normal IVD, including geometric properties and material properties, and then the parameters of the model are compared with the actual literature values or complex experimental procedures to verify the accuracy [42,43]. Solver is a core tool of FEM, which is used to analyze mechanical and thermal coupling problems. Commonly used solvers include Ansys, Abaqus, V-Biomech, and FEBio.

### Progress of FE IVD models

**Figure 4.**
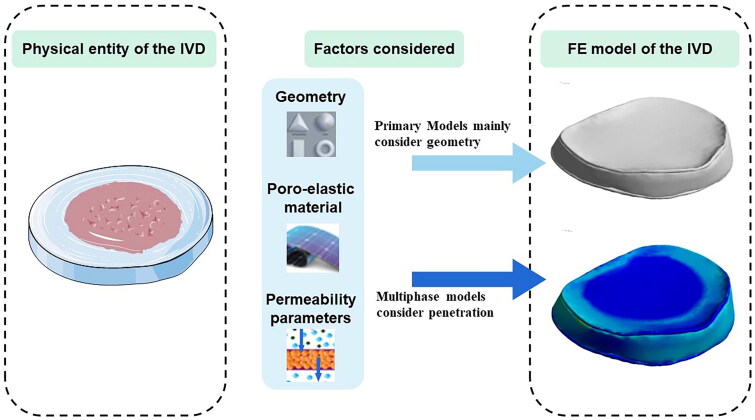
The general shape of the IVD and the characteristics of the FE models. IVD: intervertebral disc; FE: finite element.

With the advancement of technology and research, the model has developed from two-dimensional to three-dimensional, from linear to nonlinear, and to include changes in porosity and permeability. Figure 4 depicted the general shape of the IVD and the characteristics of the FE models. Image acquisition has developed into CT and MRI, improving the authenticity of the model. The mechanical response of an IVD to external loads may vary depending on the loading rate; as a result, the FE analysis of an IVD can be classified as static, quasi-static, and transient, depending on the specific research question. The biomechanics of the models are also influenced by magnitude, preloads, postures, and nucleus conditions. For example, the nucleus fluid content affects the disc compliance, and the permeability of CEP affects the time-dependent response or transport of solutes. Therefore, the FE models can also be classified as groups of elastic, multi-phasic transient, and transport models [32]. Another recent classification divides FE models into three groups: linear models, nonlinearity models of different materials and geometric, and FE models considering permeability parameters and swelling.

In this review, the FE models from the researches are divided into two categories according to the objects and methods: general models in the primary stage which mainly consider geometry and advanced multiphase models considering permeability parameters. Different materials and geometries change the characteristics and mechanical properties of IVDs under loading (Table 1). There are also multi-phase FE models made of poro-elastic materials including those with osmotic swelling (Table 2).

**Table 1.** General FE models in the primary stage.

Year	Team	Model preparation method	Model characteristics
1986	Kurowski et al. [39]	Draw finite element mesh corresponding to the anatomical shape.	Two-dimensional finite element model with co-directional material.
1996	Lu et al. [44]	Establish L2-L3 segment FE model based on CT scanning.	The change in water content is used to simulate changes of the IVD, but the model only considers fast rate load and the bending is lower than normal.
1999	Natarajan et al. [45]	Establish L3-L4 segment FE model based on CT scanning.	Perform biomechanical analysis of IVDS of different heights and areas, but only restricted to loads in daily life.
2000	Lee et al. [46]	Establish a L3-L4 segment FE model based on CT scanning.	Modify the model to incorporate poro-elastic properties and simulate the response of the segment under impact loading.
2000	Kim et al. [47]	L3-L4 segment is modeled as isoparametric elements by ANSYS.	Models are established by mechanical, geometric, and material changes with aging, but ignored muscle activation.
2014	Jacobs et al. [48]	Establish L4-L5 segment FE model based on MRI images.	Structural hyper-elastic model is used in conjunction with biphasic-swelling theory to obtain material properties.
2014	Kim et al. [49]	Modify the precious computational model and construct muscle model based on trunk muscle recruitment.	The models include more deep muscles and simulate the change in TMR in response to IVDD.
2017	Natarajan et al. [50]	Modify normal FE models to create discs of degeneration.	Degenerations are modeled through changing the height and area of IVD, but it cannot simulate living system exactly such as muscle activation.
2017	Gómez et al. [51]	Establish L3-L4 segment FE model based on CT scanning.	They combined FEM and MRS methods to obtain the material parameters and optimize the model.
2017	Lorza et al. [52]	L4-L5 segment FE model was established and parameterized.	They proposed a method to design IVD through the combination of FEM and soft computing techniques.
2018	Azian et al. [53]	Establish L4-L5 segment FE model based on CT scanning	Changes in material properties are considered, but the fluid transfer of NP and the viscoelastic characteristics of soft tissues are disregarded.
2020	Ibrahim et al. [54]	Establish models including T1-S2 based on MRI scanning acquired from a database of 3D MRI-based human body parts.	The model includes intra-abdominal pressure and structures such as intervertebral disc, vertebral body and muscles.
2021	Du et al. [55]	Established a model of fresh -frozen bovine tail based on MRI scanning.	MRI improves the accuracy of the model, but the study only considers the pure axial compression load.
2021	Liang et al. [56]	Establish six bovine IVD models based on MRI images.	Models are developed with variations of endplate geometry and NP-AF boundaries.
2022	Li et al. [57]	Establish a complete L4-S1 vertebral model of female patients, namely the isthmic spondylolysis model based on CT image segmentation technology	Six physiological motion states were simulated by applying a 500 N concentrated force and a 10Nm moment load to the four models.
2023	Xu et al. [51]	Establish a finite element model of the spine with a coupled neuromuscular model	The spinal coupling model can be used for spinal injury risk analysis caused by musculoskeletal imbalance.
2024	Muñoz et al. [58]	The calibrated structured IVD finite element (FE) model was transformed into a personalized model through a mesh morphing process.	Compared with the segmented image, the PP model obtained by this method maintains good mesh quality and integrity

***FE*** finite element, ***FEM*** finite element method, ***IVD*** intervertebral disc, ***CT*** computed tomography, ***ANSYS*** swanson analysis systems, ***IVDD*** intervertebral disc degeneration, ***NP*** nucleus pulposus, ***MRS*** multi response surface.

**Table 2.** Advanced multiphase models considering permeability parameters.

Year	Team	Model preparation method	Model characteristics
1985	Simon et al. [59]	FE model was based on fresh L2-L3 segment of rhesus monkey.	The model considers the role of the fluid phase in the mechanical response of the IVD.
1993	Laible et al. [60]	Expand the research according to the model of Simon.	The model is completely three-dimensional and considers the swelling process occurs in soft tissue.
1996	Wu et al. [61]	Establish a spinal motion segment model based on CT scanning.	The research introduces a mixed model and considers the fluid flow in the model under various loads.
2002	Riches et al. [62]	The height of IVD is determined by X-ray and the cross-sectional area was determined by technique described by Nachemson and Morris.	Use permeability parameters to study the creep pressure, and simulate the time-dependent deformation of the segment.
2007	Williams et al. [63]	Establish L4-L5 segment FE model based on CT scanning.	The model can predict the in vivo behavior and be used to study the relationship between repetitive lifting and IVDD.
2007	Soukane et al. [64]	Four regions are considered to constitute an axisymmetric model of lumbar, and the species are coupled by nonlinear equations through PH.	Consider the nutrient transport which can help understand how factors affect nutritional profiles through IVD.
2010	Chagnon et al. [65]	Establish L4-L5 segment FE model based on CT scanning.	The model incorporated the viscoelastic behavior of collagen fibers and the contribution of fluid movement.
2013	Galbusera et al. [66]	Use non-linear coupled partial differential equations to model oxygen, lactate and glucose.	The supply and removal from the capillary are modeled by the changes of oxygen, glucose and lactate.
2016	Guo et al. [67]	Establish L4-L5 segment FE model by software ABAQUS.	Material of endplates is assigned with three permeability parameters to examine the biomechanical characteristics.
2019	Derrouiche et al. [68]	Use CCD camera of DIC system to establish model of cervical IVD.	Use a chemo-mechanical approach for the stress-free swelling caused by osmosis of internal fluid content variation.
2019	Kandil et al. [69]	Consider the fluid exchange at the scale of ECM and stratification.	Establish a microstructure-based model and consider the ‘interlamellar’ ground substance between soft tissues.
2019	Yang et al. [70]	Change the content and distribution of high glycosaminoglycans to study the effect of IVDD on swelling.	Use triphasic mixture theory to describe disc tissues including a solid phase and two fluid phases.
2019	Chetoui et al. [71]	Establish two elastic biphasic models based on MRI images.	The models can predict the mechanical response of uniaxial compressive test and the redistribution of porosity after load.
2021	Castro et al. [72]	Establish a model with a new calculation in V-Biomech solver.	The model can fully reflect the natural creep of the IVD and can be used for the preparation of individualized IVD models.
2023	Fasser et al. [73]	A robust model based on the finite element method is proposed	Numerical modeling of vertebral segments in a highly elastic framework using tetrahedral elements to capture various mechanical properties.

***FE*** finite element, ***IVD*** intervertebral disc, ***CT*** computed tomography, ***TMR*** trunk muscle recruitment, ***IVDD*** intervertebral disc degeneration, ***NP*** nucleus pulposus, ***AF*** annulus fibrosus, ***CCD*** charged couple device, ***DIC*** digital image correlation, ***ECM*** extracellular matrix.

#### General FE models in the primary stage

The FE model has been used to study the relationship between the degeneration of IVD and the changes in biomechanical characteristics. Kurowski et al. [39] used two-dimensional FE to study the load transfer mode, stress concentration, and the most likely diseased part of the IVD. Directional homogeneous materials were used to simulate the AF and static water was applied to the AF wall. The pressure was used to simulate the NP, and the degeneration of the IVD was simulated by the reduction of the hydrostatic pressure in the NP. In this study, the degeneration model of the IVD only represents the initial stage when the NP is like fluid and not the mature stage of the degeneration process. This simplified model shows that the cracks extend from the inside to the surface of the cortical shell during mild disc degeneration. Although it failed to show the early changes in the NP during the degeneration process, the study elucidated that the initial stage of the degeneration occurred in the endplate area. Lu et al. [44] used a viscoelastic FE model to study the occurrence and development of the annulus degeneration under axial compression and buckling loads, simulated the AF with viscoelastic materials, and simulated the diurnal hydrostatic pressure of the NP with various water contents. The CEP and anulus ground substance were simulated by 3D 8-node brick elements and 3D hydrostatic fluid elements were used for NP. The results showed that degeneration of the AF began at the connection between the inside of the AF and the endplate. The weakest part of the IVD is also the earliest degeneration part because it bears the combined effect of the buckling load and axial compression load. To study the lateral behavior of the AF, Natarajan et al. [45] applied different loads and directions to the FE model to study the occurrence and development of IVDD. 20-node isoparametric elements were used to model IVD and they used an iterative method to determine the highest stress positions of the endplate and AF. The degeneration of the IVD started from the endplate, further proving that the extension has a greater impact on the IVDD than other positions.

Lee et al. [46] improved upon Simon’s research and studied the impact of a sudden load on the motion segments of the lumbar spine. The IVD was modeled as composed of 20-node brick elements and 8-node shell elements. The results showed that the vertebral body fractures are related to the instantaneous impact load, which are most likely to start from the endplate or posterior wall of the cortical bone. Kim [47] used a young spine model and an old spine model to study the mechanical behavior of aged-related degeneration of human spine segments. He modeled the NP as solid elements with different Young’s modulus for the young and old model and the AF was modeled with 8-node laminate solid elements. The experiment showed that the compressive strain is in the posterior anulus and the interlaminar shear stresses increase with the rupture around the AF. Jacobs et al. [48] used high-resolution MRI images to establish an FE model to verify the change of material properties in the mechanical response of the lumbar IVD without calibration. The 3D mesh was composed of 8-node hexahedral elements and a new set of biphasic-swelling material parameters was used with a consistent protocol. Kim et al. [49] established a degeneration model using a previous model and analyzed the difference between the degeneration of the IVD and the change in muscle strength by reducing the volume and elastic modulus of the IVD. The results showed that the degeneration of the IVD reduced the activity of the deep muscles and increased the activity of the superficial muscles. Therefore, the pressure inside the IVD is reduced and the speed of the degeneration of the IVD is slowed. These differences are expected to be functional because they reduce stress on the degenerating disc by altering muscle activation. This model is therefore very relevant to help prevent risk factors and slow the progression of disc degeneration. Natarajan et al. [50] established lumbar IVD models with different degrees of degeneration by changing the height and area of the IVD, and found that the severity of the degeneration of IVD, the number of degenerations, and the degenerated segments will affect the movement of adjacent discs, and then lead to degeneration of the adjacent discs. Azian et al. [53] constructed a functional three-dimensional non-linear FE model to study the effect of material degeneration of the IVD tissue on the mechanics of the lower lumbar spine unit, and simulate different factors by introducing degeneration factors into the material properties. The IVD was modeled by 8-node second-order hexahedral elements. The degree of IVDD, comparing the spinal movement and intradiscal pressure of healthy and degenerated IVDs with the data *in vitro*, verifies the validity of the model and elucidates that the existence of IVD pressure increases spinal flexion and axial rotation instability. To improve the effectiveness and comprehensiveness of the model, Ibrahim et al. [54] proposed a new FE meshing method that eliminated all contacts computations, and successfully constructed and verified a new three-dimensional FE model. The model includes intra-abdominal pressure and anatomical structures, such as the IVD, vertebral body, abdominal wall, muscles, and tendons, which lay the foundation for further research on degenerative disc diseases. Du et al. [55] established 6 FE models of different IVD shapes using MRI, and performed biomechanical analysis. The results showed that the stiffness of the IVDs with complex shapes is 3.1 times greater than that of the simply shaped IVDs. Therefore, the authenticity of the model’s geometry should be ensured when modeling, thereby improving the accuracy of the load distribution of the IVD. Liang et al. [56] used isotropic materials to establish a FE model containing IVDs and the spinal cord, and then simulated the influence of different degrees of disc herniation on the spinal cord. The IVD was meshed with 8-node hexahedral elements and 1 mm element size verified the accuracy of the FE model. The results showed that the size and position of the herniated disc varies with the change in temperature, size, and position of the expansion element. The deformation and stress distribution of the spinal cord increase with the increase in the degree of expansion. This method can be further used for the individualized research of spondylotic myelopathy.

Li et al. reconstructed a complete model of the L4-S1 vertebra of a female patient, a spondylolysis model, a spondylolysis model with PSUSR internal fixation, and a spondylolysis model with PSVPH internal fixation by using CT image segmentation. Six physiological motion states were simulated by applying a 500 N concentrated force and a 10Nm moment load to the four models. The range of motion (ROM), maximum stress, maximum strain, and maximum displacement of the models were compared. The results showed that the PSVPH internal fixation system had better biomechanical properties than the PSUSR internal fixation system [57]. Xu et al. established a finite element model of the spine with active muscles around the vertebrae, verified it from anatomical verification to the whole spine model under multi-level dynamic loading, and coupled the neuromuscular model to quantitatively analyze the effects of erector spinae (ES) and multifidus (MF) sarcopenia on spinal tissue. The results showed that sarcopenia mainly led to high injury risks in L4-L5 intervertebral discs, L1 vertebrae, and L3-S1 joint capsules in terms of significant pressure or strain variations. The spinal coupling model can be used for spinal injury risk analysis caused by musculoskeletal imbalance [51]. Muñoz et al. applied a novel approach to transform a calibrated structured IVD finite element (FE) model into 169 patient-personalized (PP) models through a mesh morphing process. The true shapes of the patient’s annulus fibrosus (AF) and nucleus pulposus (NP) were accurately replicated while maintaining the same topology for all models. The method achieved PP models with 92.06% and 92.10% similarity for AF and NP, respectively, compared to the segmented images. The models maintained good quality and integrity of the mesh. The cartilaginous endplate (CEP) shape was rendered at the IVD-vertebra interface, ensuring mesh personalization. The influence of local morphology on the indirect mechanotransduction response was revealed, highlighting the role of height, sagittal area, and volume. While the maximum principal stress was affected by morphology such as height, the ellipticity of the IVD affected the minimum principal stress. The results showed that the CEP was not affected by its local morphology but by the AF and NP [58].

Gómez et al. proposed a method to determine the relationship between BMI and functional spinal unit [52]. During the construction of FE model, 8-node hexahedral solid elements formed the NP and homogenous ground substance, whereas two-node link FE elements simulated the fibers. The type of size for modeling was based on the previous literature and the multi-response surface method combined with FEM was used to optimize the model [74]. They also proposed an automated method to determine the material parameters based on experimental data. Based on evolutionary optimization techniques, the regression models with the best generalization capacity were used to set the parameters in IVD [75]. Similarly, Lorza et al. combined FEM and soft computing techniques to simulate the compression and lateral bending stiffness from the artificial IVD [76].

#### Advanced multi-phase models

Changes in the distribution of nutrients and metabolites, as well as the changes in the solute penetration parameters in the NP, will cause degeneration of the IVD, impacting the height and biological characteristics of the IVD. Therefore, the changes in the penetration parameters of the IVD should be fully considered when modeling. Simon et al. [59] first used the theory of poro-elastic media in the modeling of IVDs. In the process of modeling, the solid phase of IVD was simulated with a porous solid that can be deformed, and the liquid phase of the IVD was simulated with the liquid flowing in the pores. Considering the elastic properties of the IVD and the compressibility, permeability, and porosity of the solid and liquid phases, the results show that the volume of the IVD is reduced after the load is removed, and the disc is rehydrated after the load is removed. The destruction is related to the destruction of the cancellous bone near the endplate because this degeneration changes the fluid flow pattern and nutrient pathways. Decreased disc height, increased disc bulge, altered fluid motion, and stress were quantified in the model developed in this study. Making the model data more realistic and simulating processes such as disc degeneration will be more instructive. Wu et al. [61] established a similar model. They also took the rear structure into account, and their conclusion was similar to Simon’s long-term creep load. Since none of the above models include the expansion pressure of the IVD, it is impossible to predict the return of fluid when the load is removed. Laible et al. [60] considered the swelling of the soft tissue when establishing the porous FEM. The expansion pressure reduced the load of the solid phase and increased the load of the liquid phase at the same time, thereby enhancing the segmental load resistance. Riches et al. [62] constructed a poro-elastic model using permeability parameters as a strain function to study the creep and expansion pressure of the IVD, and simulate the time-dependent deformation of the vertebral body motion segment. Williams et al. [63] established a poro-elastic FE model to study changes in the fluid volume of the NP and the interaction between fluid and proteoglycans. Proteoglycans in the NP are the driving force for fluid in the surrounding tissues to flow into the IVD and resist the outflow of the IVD. When the fluid flows, the expansion pressure is caused by the change in the fixed charge density of the NP. The measured height change of the lumbar IVD is compared with the results obtained in this study to verify the validity of the model. The model was then used to predict the changes in the biomechanical behavior of the lumbar IVD caused by the degeneration of the IVD. Soukane et al. [64] found that the content of lactic acid gradually increased from the CEP to the center of the NP, and this trend became more obvious as the height of the IVD increased with the permeability of the cartilage.

Chagnon et al. [65] used 3D 8-node elements to represent the model and set NP area at 40% of the disc cross-sectional area and translated by 3 mm posteriorly. They found that the AF and NP creep one after another under a payload, and the osmotic pressure of the NP affects the stress distribution of the IVD and solute transport. Galbusera et al. [66] showed that the concentration of oxygen and glucose in the IVD decreases with the increase in the distance from the supply source to the outer ring of the NP. The changes in the endplate porosity, cell number, consumption rate, and diffusion rate significantly affect the concentration of glucose and oxygen, thereby affecting the osmotic pressure in the NP. Yang et al. [70] studied the effect of IVDD on swelling by changing the content and distribution of glycosaminoglycans, and discussed that directly changing the biochemical composition and distribution of the IVD can simulate changes in the geometric shape of the IVD. In their model, the NP explant geometry was a 1 mm cube and meshes for AF included 20 individual lamellae with 109K elements. The lamellae and subcomponents were welded together by sharing nodes.

The FE models for IVD have been further developed with the widespread use of MRI and solvers. Guo et al. [67] analyzed the deformation, stress distribution, and fluid flow of the IVD using a poro-elastic FE model with different permeability parameters. The IVD and vertebral bodies were modeled with 20-node reduced integral quadratic element. Their results showed that the pore pressure of the disc gradually decreased under intermittent compressive loads, and the effective stress gradually increased. In the process of removing the load, the greater the permeability of the CEP, the faster the flow of fluid back to the IVD. Chetoui et al. [71] used MRI to determine the geometry and porosity of the IVD, and established two biphasic models, isotropic and anisotropic that is continuous in space, with elastic extracellular matrix. The mesh convergence study was performed to reduce the dependence of mesh density for computing unknowns and finally the tetrahedral elements were used. Based on the analysis of the differences between different IVD models, both models can simulate the changes in porosity after loading. Fasser et al. [73] proposed a robust model based on the finite element method, using tetrahedral elements, and numerically modeling vertebral segments in a highly elastic framework to capture various mechanical properties and better address the challenges of numerical modeling of intervertebral discs. The stability and accuracy of the model were evaluated through numerical simulations, with a special focus on the degenerative disc and its possible distortion and narrowing geometric features. The model used fiber-reinforced Mooney-Rivlin type solids to simulate the annulus fibers. An adaptive state variable-dependent explicit time step was proposed and applied as a computationally efficient alternative. It was found that the tetrahedral element-based model provided greater stability and flexibility in the geometric mesh representation and study of various degenerative stages of the spinal segment compared to the commonly used hexahedral elements. Therefore, the FE analysis of the IVD models under different loads may be an effective tool to assess early disc degeneration. Therefore, the FE analysis of the IVD models under different loads may be an effective tool to assess early disc degeneration. Derrouiche et al. [68] proposed a new chemical–mechanical method to study the inherent permeability–inelastic characteristics of the AF. When constructing the model, 8-node meshing elements, isoparametric and arbitrarily hexahedrics were used for meshing and the inelastic stress of the extracellular matrix and swelling caused by the liquid flow under the osmotic action were fully considered. Permeation–inelastic coupling is a reversible process that depends on the fluid exchange between the AF layers, revealing that the microstructure of the AF plays an important role in the model building process. However, the complex interaction between intrinsic properties and fluid transfer was depend on the parameters of the model and the morphological features of the annulus. This resulted in unavoidable discrepancies between the model and the actual IVD. Kandil et al. [69] used different materials to simulate the physical and chemical properties of the AF, establishing a microstructure-based FE model containing the interlaminar cell matrix of the annulus. Three-dimensional 8-node meshing elements, isoparametric and arbitrarily hexahedrics were used for meshing, and the element size is independent to the mesh. Then they used the free energy function to study the fluid flow and fluid flow inside the IVD. The viscoelasticity of the extracellular matrix was able to simulate the lateral behavior under the microstructure of the IVD. The stiffness of the IVD varies greatly in different loading directions because of the existence of intervertebral joints. Although the incidence of spinal traction injuries, such as cervical whiplash injury cannot be ignored, there are still few studies on the tensile stiffness of the IVD.

Castro et al. [72] established a porous viscoelastic FE model that implemented an existing formulation in their solver named V-Biomech. The linear 4-node tetrahedron, the quadratic 10-node tetrahedron and the quadratic 27-node hexahedron were used for modeling. The effectiveness was verified, and the results showed that the model can reflect the natural creep of the IVD and can be used for preparing individualized IVD models.

We have described several FEM models showing different types of modeling parameters, including mechanical features and geometric dimensions. Once these parameters and their associated effects are determined, a more accurate FEM model can be built by measuring/modeling the important sensitive parameters while ignoring the less important parameters. Cappetti et al. [77] developed a fast procedure based on the finite element/Taguchi method. Through this simple procedure, many relevant parameters can be optimized and simulated by detailed modeling of the most influential areas characterized by sensitive features, making the established FEM model more accurate.

### Limitations and prospects

IVD FE models have advantages in terms of accurately simulating the shape and biomechanical properties. Alternatively, the FE models can simulate very complex tissue structures or load systems, and help us understand the relationship between the biomechanical properties and degeneration of the IVD. FE models can also optimize the clinical operation plan and predict the long-term effect of the operation by analyzing the mechanical changes of the IVD under various load conditions. However, IVD FE models have some limitations. First, an FE model only represents a numerical model, which can only be approximated by samples or obtained directly from actual conditions, not specifically obtained through anthropometric measurements or medical image data [78]. Second, the FEM of the IVD is restricted by complex anatomical factors, such as the non-linear characteristics of the surrounding skeletal muscles, tendons, and ligaments, which are difficult to use and to achieve accurate simulation, especially the dynamic changes in the size and direction of the muscles [79]. A lot of in-silico studies are based on animal discs. Since human discs are quite different from animal discs, researches should address the validity of animal IVDs as a model of human discs. Another important issue is that current FE models do not include cell behavior, which is affected by mechanical loading as a clear consensus. There is plenty of experimental evidence that mechanical loading affects the metabolism and inflammatory of cells, which has not been clarified in a FE model yet [80–82]. Furthermore, the current FEM is based on the quasi-static force analysis of different attitudes and the separating of the actions, which is difficult to realize the real-time dynamic response of the physical object. The analysis of individualized biomechanical properties is also a difficulty due to the great differences in the biomechanical properties of bones in different individuals and different genders. In addition, the processing of various parameters of the FE model may increase the possibility of error.

A hybrid model combining AI algorithms and simulation will become more popular [83]. To develop an accurate IVD model for a patient IVD, it is necessary to explore the individual design of the multiphase model in combination with clinical cases. Based on the collected multi-dimensional data, the virtual IVD is perfectly replicated through modeling, including appearance and size, muscles, and bones; the virtual IVD displays human biochemical indicators at multiple spatial scales of tissues, cells, and molecules. Then, more works are needed to make a reliable connection between multi-scale models and microstructures. Moreover, another suggestion could be that regenerative strategies require a better constitutive representation of degenerative features and that the IVD mechanical parameters (bulk modulus, permeability, etc.) need to be tuned quantitatively in order to obtain a functional replacement. It would also be necessary to consider the responses under more complex mechanical loading conditions. The physiological parameters combined with fluid mechanics, elastic mechanics, physiological and pathological operating rules of organs or tissues consciously or unconsciously controlled by the nerve center should be considered. Additional studies simulating the real human IVD with an FE model, improving the integration of medical and engineering disciplines, and improving the level of clinical research and innovation are needed to provide the scientific experimental basis for the incidence, development, treatment, and prognosis of IVD-related diseases.

## Conclusions

Changes in biomechanical properties have an important role in the incidence and development of IVDD. FEM is an important tool in biomechanical research and FE models have been developed rapidly with the application of technologies. An outstanding advantage of a FE model is that the computer can simulate and analyze its impact on the final result by changing any parameter. However, existing models still have disadvantages in terms of accuracy and personalization. The imaging data of the spine and the combination with a parametric model can be used to establish a detailed virtual IVD in the information world as a complex system for research. In order to explore the individual and synergistic impacts of the major components regulating the overall mechanics, in-silico studies built by machine learning and creating more trustable linkages with the IVD multiscale structure under increasingly complicated mechanical loading circumstances should be used. In clinical terms, the biomechanical analysis of the IVD model can simulate and predict the true response of a patient’s IVD to stress and the microenvironment under different conditions, providing a theoretical basis for precise medical treatments. In the future, consider establishing reliable connections between the multi-scale models and microstructures under more complex mechanical loading conditions. This would help establish more accurate FE models of the IVD, thereby laying a theoretical foundation for the realization of precision medicine.
